# Inhibitory effect of Carnosol on UVB-induced inflammation via inhibition of STAT3

**DOI:** 10.1007/s12272-018-1088-1

**Published:** 2018-11-15

**Authors:** In Jun Yeo, Ju Ho Park, Jun Sung Jang, Do Yeon Lee, Jung Eun Park, Ye Eun Choi, Jung Hun Joo, Joo Kyung Song, Hyung Ok Jeon, Jin Tae Hong

**Affiliations:** College of Pharmacy and Medical Research Center, Chungbuk National University, 194-31 Osongsaengmyeong 1-ro, Osong-eup, Heungduk-gu, Cheongju, Chungbuk 361-951 Republic of Korea

**Keywords:** Carnosol, Dermatitis, STAT3, UVB

## Abstract

Ultraviolet B (UVB) irradiation causes sunburn, inflammatory responses, dysregulation of immune function, oxidative stress, DNA damage and photocarcinogenesis on skin. Rosemary (*Rosmarinus officinalis* L.) has been reported to inhibit inflammation. Carnosol, a major component of Rosemary, has prominent anti-inflammatory effects. However, its protective effect on UVB-induced inflammatory skin responses has not yet been reported. Here, we investigated the effectiveness of carnosol on UVB-induced inflammation. We examined the anti-inflammation effect of topical application of carnosol (0.05 µg/cm^2^) on UVB (540 mJ/cm^2^, for 3 successive days)-induced skin inflammation in HR1 mice. Topical application of carnosol inhibited UVB-induced erythema, epidermal thickness, inflammatory responses in HR1 mice. Carnosol reduced the level of Immunoglobulin-E and IL-1β in blood serum of UVB-induced mice. Carnosol also significantly inhibited the UVB-induced expression of inflammatory marker protein (iNOS and COX-2) in back skin of mice. In addition, carnosol treated skin decreased activation of STAT3, a transcriptional factor regulating inflammatory genes. Our study suggested that carnosol has protective effects on skin inflammatory skin damages by UVB.

## Introduction

UVB-irradiation is one of the most dangerous environmental factors causing several pathologic changes such as sunburn, erythema, edema, and skin cancer (Baek et al. 2017). One of the major deleterious outcomes on the skin is the production reactive oxygen species (ROS) that contribute to cause cellular damages (Bickers and Athar 2006; Van Laethem et al. 2009). UVB irradiation induces skin oxidative stress deplete antioxidant defenses such as reduced gluthathione (GSH) and superoxide dismutase (Hasegawa et al. 1992). UVB irradiation also induces skin damages through the production of inflammatory mediators (Afaq 2011; Oresajo et al. 2012). ROS can induce pro-inflammatory mediators, thus cause skin damages after UVB exposure (Casagrande et al. 2006; Ivan et al. 2014).

STATs constitute a family of cytoplasmic proteins that play critical roles in transmitting signals from extracellular stimuli to the nucleus in normal cells (Darnell 1997; Levy and Darnell, 2002; Yu et al. 2002). Activation of STAT3 is important for the development of atopic dermatitis, thus, several anti-inflammatory compounds such as quercetin that can inhibit the development of atopic dermatitis by preventing STAT3 activation (Karuppagounder et al. 2016). STAT3 is also involved in IgE dependent mast cell degranulation in the human and mice skin (Siegel et al. 2013). In inflammatory skin lesions, expression and activation of STAT3 has been documented, and in normal human keratinocytes, IFNs and IL-6 induce STAT3 activation (Andres et al. 2013). It is demonstrated that phosphorylated STAT3 may be a therapeutic target (Takeichi et al. 2011). STAT3 is critical for cytokines induced synovial infiltration in inflammatory skin disease (Nowell et al. 2009). Activation of STAT3 is also involved in skin barrier formation (Amano et al. 2015). Thus, compounds inhibiting STAT3 could be effective for atopic dermatitis.

Several phytochemicals are important group of drug like agents since they have low toxicities and benefit for several diseases (Chung et al. 2007). Rosemary (*Rosmarinus Officinalis L.*) is an aromatic evergreen herb native to the Mediterranean region, which is an important component of the Mediterranean diet, and has been used in traditional medicine. Modern pharmacological studies have demonstrated that rosemary extract has anti-oxidant (Santoyo et al. 2005), anti-inflammatory (Bozin et al. 2007), and anti-cancer activity (Atsumi and Tonosaki, 2007). Previous studies demonstrated that carnosol, one of components of rosemary extract, significantly inhibited inflammatory responses such as TNF-α, IL-1β, and IL-10 generation (Yao et al. 2014; Schwager et al. 2016), NO generation, and expression of iNOS and COX-2 in inflamed mice skin (Mengoni et al. 2011). However, its protective effect on UVB-induced atopic inflammatory responses has not been reported yet. In the present study, we investigated anti-inflammatory and anti-dermatitic effects of carnosol extracted from rosemary leaves in UVB-exposed atopic dermatitis mice.

## Materials and methods

### Ethical approval

The experimental protocols were carried out according to the guidelines for animal experiments of the Institutional Animal Care and Use Committee (IACUC) of Laboratory Animal Research Center at Chungbuk National University, Korea (CBNUA-929-16-01). All efforts were made to minimize animal suffering, and to reduce the number of animals used. HR1 mice were housed in three mice per cage with automatic temperature control (21–25 °C), relative humidity (45–65%), and 12 h light–dark cycle illuminating from 08:00 a.m. to 08:00 p.m. Food and water were available ad libitum. They were fed pellet diet consisting of crude protein 20.5%, crude fat 3.5%, crude fiber 8.0%, crude ash 8.0%, calcium 0.5%, phosphorus 0.5% per 100 g of the diet (collected from Daehan Biolink, Chungcheongbuk-do, Korea). During this study, all mice were specially observed for the normal body posture, piloerection, ataxia, urination, etc. 2 times per day.

### Animal treatment

UVB irradiation source consisted of a Philips TL40 W/12 RS lamp (Medical-Eindhoven, Holland) mounted 20 cm from mice. It emitted a continuous light spectrum between 270 and 400 nm with a peak emission at 313 nm. UVB output (80% of total UV irradiation) was measured using an IL-1700 model Research Radiometer (International Light, USA; calibrated by IL service staff) with a radiometer sensor for UVB (SED240). Mice were anesthetized with a single intraperitoneal injection of 90 mg/kg of ketamine plus 3 mg/kg of xylazine followed by exposure to UVB irradiation at 540 mJ/cm^2^. Both ear and back skin of each animal were exposed to UVB irradiation for 15 min each day for three consecutive days. One hour before UVB irradiation, 100 μl (20 μl/cm^2^) of 10 µM of Carnosol in 0.05% dimethyl sulfoxide (DMSO) was applied to the dorsum of ears and back skin three times a week for four weeks as treatment group. For the control group, 100 μl of 0.05% DMSO was applied.

### Measurement of body and lymph node weight, and ear thickness

Alterations of body weight during the experimental period were measured with an electronic balance (Mettler Toledo, Greifensee, Switzerland) once a week for 4 weeks. Weights of lymph nodes collected from sacrificed mice were measured with the same method. Ear thickness was measured using a thickness gauge (Digimatic Indicator, Matusutoyo Co., Tokyo, Japan) to determine the degree of allergic skin inflammation induced by UVB treatment.

### Histological techniques

Ear and back skins were removed from mice, fixed with 10% formalin, embedded in paraffin wax, routinely processed, and then sectioned into 5 μm thick slices. The skin sections were then stained with hematoxylin and eosin (H&E). The thickness of the epidermis and dermis were also measured using the Leica Application Suite (Leica Microsystems, Wetzlar, Germany).

### Enzyme-linked immunosorbent assay (ELISA) for detection of serum IgE concentration

Serum IgE concentration was measured using an ELISA kit (Shibayagi, Inc., Gunma, Japan) according to the manufacturer’s instructions. Briefly, capture antibodies were added into Nunc C bottom immunoplate supplied bu the kit. Next, wells were washed with washing solution (50 mM Tris, 0.14 M NaCl, 0.05% Tween 20, pH 8.0) three times. Serum samples and standards diluted with buffer solution were then added to wells and the plate was incubated for 2 h at 37 °C. Wells were then washed with washing solution and 50 μl of biotin-conjugated anti-IgE antibody (1000-fold dilution) was added to each well followed by incubation at room temperature for 2 h to bind with captured IgE. Wells were washed again with washing solution, and then horseradish peroxidase-conjugated detection antibody (2000-fold dilution) was added to each well and incubated at room temperature for 1 h. An enzyme reaction was then initiated by adding tetramethylbenzidine (TMB) substrate solution (100 mM sodium acetate buffer pH 6.0, 0.006% H_2_O_2_) to each well followed by incubation at room temperature in the dark for 20 min. Finally, the reaction was terminated by adding acidic solution (reaction stopper, 1 M H_2_SO_4_) and the absorbance (yellow product) of each well was measured spectrophotometrically at wavelength of 450 nm. The final concentration of IgE was calculated.

### Cytokine assay

By the end of the study period, blood specimens were collected. Serum levels of mouse TNF-α and IL-1β were measured by enzyme linked immunosorbent assay (ELISA) using kits obtained from Thermo Fisher Scientific (Rockford, IL, USA) according to the manufacturer’s protocol.

### Blood cell number measurements

Mice blood was taken by heart puncture. Blood cell number were measured by an automatic hematologic analyzer ADVIA2120 (Siemens Healthcare Diagnostics) in the laboratory animal research center at Chungbuk National University.

### Western blot analysis

Skin or ear tissues (100 mg) were homogenized with lysis buffer [50 mM Tris pH 8.0, 150 mM NaCl, 0.02% sodium azide, 0.2% SDS, 1 mM phenyl methylsulfonyl fluoride (PMSF), 10 μl/ml aprotinin, 1% igapel 630 (Sigma Chem. Co. St. Louis, MO, USA), 10 mM NaF, 0.5 mM EDTA, 0.1 mM EGTA and 0.5% sodium deoxycholate]. Homogenates were centrifuged at 23,000 g for 1 h. Equal amounts of protein (20 μg) were separated on sodium dodecyl sulfate (SDS)/10%-polyacrylamide gels and then transferred to nitrocellulose membranes (Hybond ECL, Amersham Pharmacia Biotech Inc., Piscataway, NJ, USA). Blots were blocked for 2 h at room temperature with 5% (w/v) non-fat dried milk in Tris-buffered saline [10 mM Tris (pH 8.0) and 150 mM NaCl] containing 0.05% Tween-20. Membranes were incubated with specific antibodies at room temperature for 4 h. Rabbit polyclonal antibodies against iNOS and COX-2 (1:500), and Rabbit monoclonal antibodies for JAK2 and p-JAK2, and IL-1β (1:500) (Santa Cruz Biotechnology Inc. Santa Cruz, CA, USA) were used in study. Mouse polyclonal antibody against STAT3, and mouse monoclonal antibody for TNF-α (1:500) (Santa Cruz Biotechnology Inc. Santa Cruz, CA, USA) were used. Blots were then incubated with corresponding horseradish peroxidase-conjugated anti-rabbit immunoglobulin G (Santa Cruz Biotechnology Inc. Santa Cruz, CA, USA). Immunoreactive proteins were detected with enhanced chemiluminescence (ECL) western blotting detection system.

### Electromobility shift assay

Gel mobility shift assay was conducted using a slight modification of a previously described method (Lee et al. 2011). In brief, 10 μg of nuclear protein of skin tissue was incubated in 25 μl of total volume of incubation buffer (10 mmol/l Tris, pH 7.5, 100 mmol/l NaCl, 1 mmol/l dithiothreitol, 4% glycerol, 80 mg/l salmon sperm DNA) at 4 °C for 15 min, and then incubated with 9.25 mBq [γ-32P] ATP-labeled oligonucleotide containing the STAT3 binding site (TCGTTCGATTCCGGGAATTGA) at room temperature for 20 min. The DNA–protein binding complex was electrophoretically resolved on a 6% nondenatured polyacrylamide gel at 150 volts for 2 h, and then the gels were dried and autoradiographed using Kodak MR film at − 80 °C overnight.

### Statistical analysis

All experiments were conducted in triplicates. All experiments were repeated at least three times with similar results. All statistical analyses were performed using GraphPad Prism 5 software version 5.03 (GraphPad software, Inc., San Diego, CA, USA). Group differences were analyzed by one-way analysis of variance (ANOVA) followed by Tukey’s multiple comparison test. All values are presented as mean ± SD. Significance was set at *P* < 0.05 for all tests.

## Results

### Effects of carnosol treatment on ear thickness and morphology

**Fig. 1:**
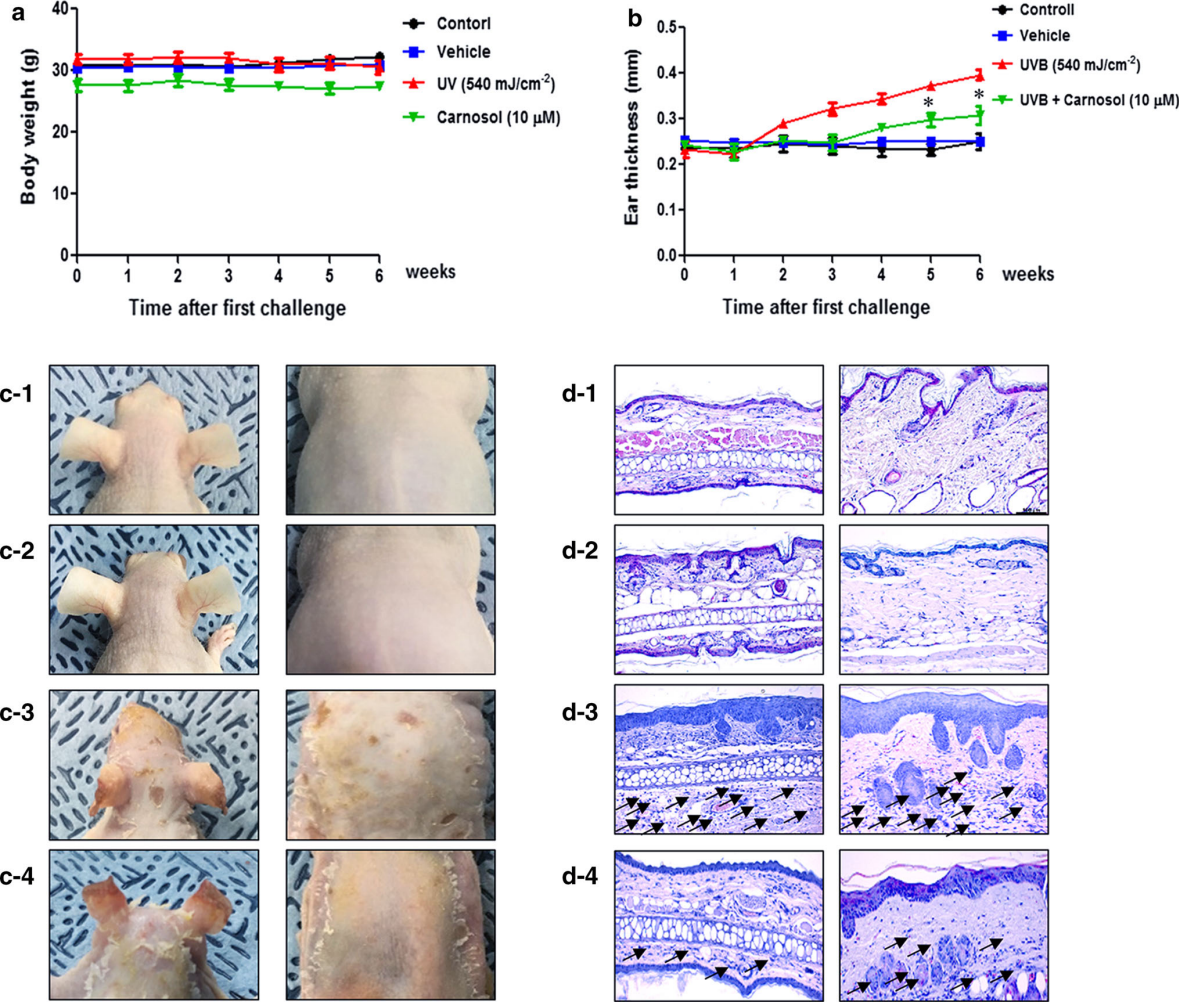
Differences in body weight, ear thickness, ear and back phenotypes and histology. UVB irradiation (540 mJ/cm^2^, 15 min a day for three successive days) was exposed during topical application of carnosol (0.05 µg/cm^2^). After 4 weeks, body weight (**a**) and ear thickness (**b**) were observed at least three times following the procedure described in Materials and Methods. *P < 0.05, significant difference compared to UVB treated group. Phenotypes (**c**) of mouse randomly selected from each group (1 mouse/group). Histopathology of ear and back skin in control (**d-1**), vehicle (**d-2**), UVB (**d-3**), and UVB + carnosol (**d-4**). Histopathological changes in the slide sections of ear and back tissue were identified by staining with hematoxylin and eosin followed by observation at × 200 magnification. Scale bars, 100 μm. Data shown are mean ± SD (n = 10)

Changes in body weight were measured during the experimental period. No significant change in body weight was detected after any treatment (Fig. 1a). To investigate whether treatment with carnosol could suppress changes in ear phenotype induced by UVB, ear and back skin thickness and morphology of ear were observed. Ear thickness was rapidly increased in UVB exposed mice compared to that in control or vehicle group of mice. On the other hand, ear thickness in carnosol treated mice was slightly decreased (Fig. 1b). Erythema, edema, and erosion were observed in UVB exposed group, but not in the control or vehicle group. These changes of ear and back morphology, and ear thickness were dramatically inhibited by carnosol treatment (Fig. 1c). To investigate the suppressive effect of carnosol treatment on ear and back histology, histological analyses of the ear and back skin were performed (Fig. 1d). There were significant increases of thicknesses of epidermis and dermis and infiltration of inflammatory cells in UVB exposed group compared to those in the control group. These changes of ear and back histology were also inhibited upon carnosol treatment (Fig. 1d).

### Effect of carnosol treatment on the release of inflammatory cytokines and IgE concentration

**Fig. 2:**
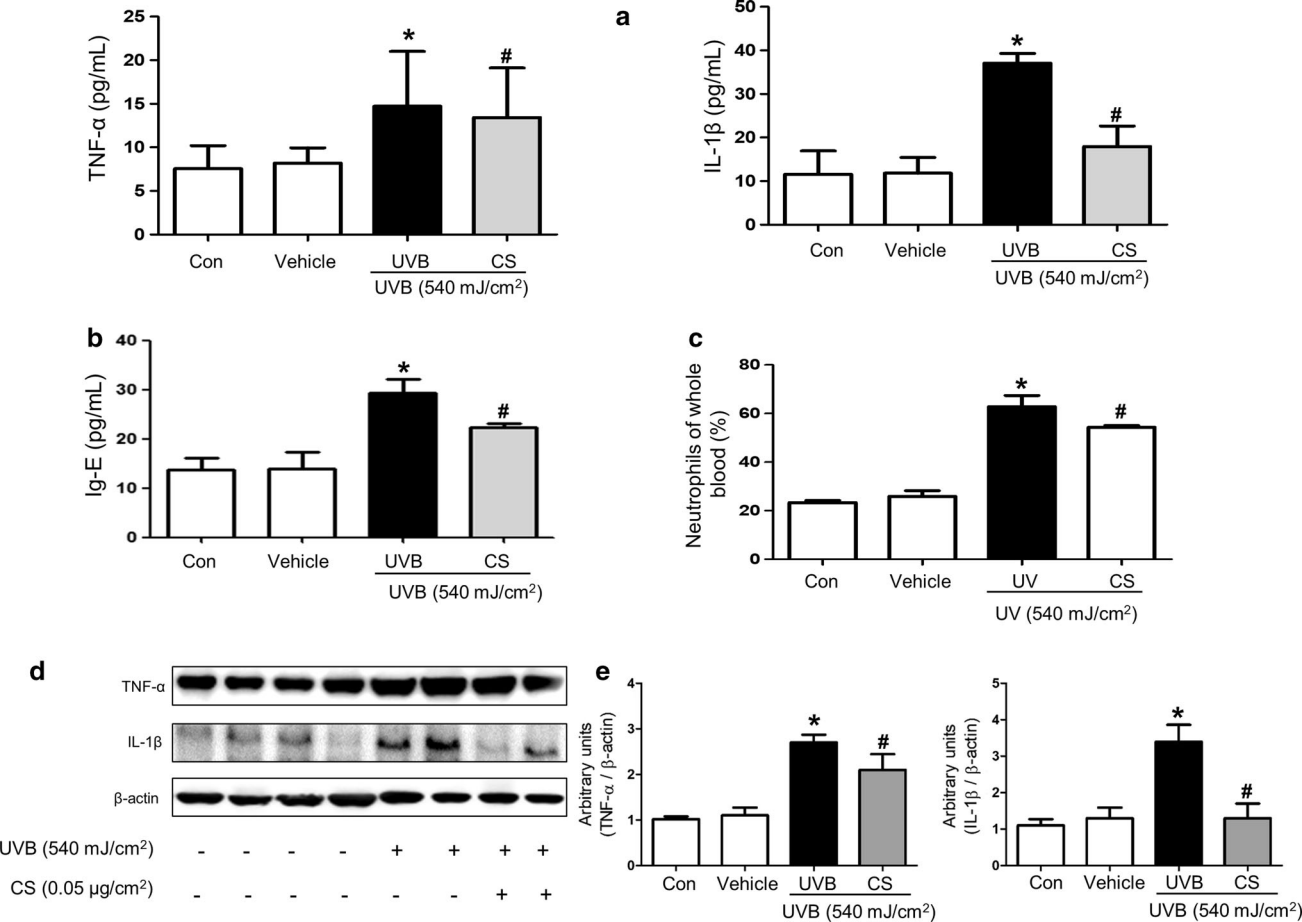
Changes in serum cytokine concentration, and expression of TNF-α, IL-1β in the back skin. After final treatment, mice from each group were sacrificed under anesthesia. Serum was used to measure cytokine concentration. It was prepared from blood sample collected from the abdominal vein of each mouse. Serum levels of TNF-α and IL-1β (**a**), IgE (**b**), were quantified by ELISA. Data shown are mean ± SD (n = 10). Blood cell (**c**, Neutrophile) number was measured by an automatic hematologic analyzer ADVIA2120 (Siemens Healthcare Diagnostics) in the laboratory animal research center of Chungbuk National University. *P < 0.05, significant difference compared to the control group. ^#^*P *< 0.05, significant difference compared to UVB exposed group. Protein expression levels of TNF-α, and IL-1β in the back skin (**d**) were measured by Western blotting. Relative densities of protein bands were quantified (**e**) following the procedure described in Materials and methods. Equal amounts of total proteins (20 μg/lane) were subjected to 10% SDS-PAGE and expression levels of TNF-α and IL-1β protein were detected by Western blotting using specific antibodies. β-actin protein was used as an internal control. Data shown are mean ± SD (n = 2). *P < 0.05, significant difference compared to the control group. ^#^*P *< 0.05, significantly different compared to UVB exposed group

To determine if carnosol treatment could induce alterations in release of inflammatory cytokines in UVB-induced skin inflammation, serum levels of TNF-α and IL-1β were measured for control, vehicle, UVB, and UVB + carnosol treated group of mice. Levels of TNF-α and IL-1β in UVB exposed group were generally higher than those in the control or vehicle group. However, these levels in carnosol treated group were dramatically decreased to levels in the control or vehicle group (Fig. 2a). Serum IgE concentration was measured to determine whether carnosol suppressed allergic responses induced by UVB. UVB exposure induced significant increase in serum IgE concentration. However, significant lower level of IgE concentration was observed in carnosol treated group (Fig. 2b). Moreover, the number of neutrophils in whole blood was higher in UVB exposed group than that in the control or vehicle group. However, it was decreased in carnosol treated group (Fig. 2c). We also investigated whether carnosol could suppress expression of inflammatory genes; TNF-α and IL-1β in ear and back skin tissues by Western blot analysis. Agreed with the serum levels, expression levels of TNF-α and IL-1β in ear and back skin tissues were significantly increased in UVB exposed mice, but carnosol decreased the increase by UVB in the expression of TNF-α and IL-1β (Fig. 2d and 2e).

### Effect of carnosol treatment on expression of iNOS and COX-2 in lymph node

**Fig. 3:**
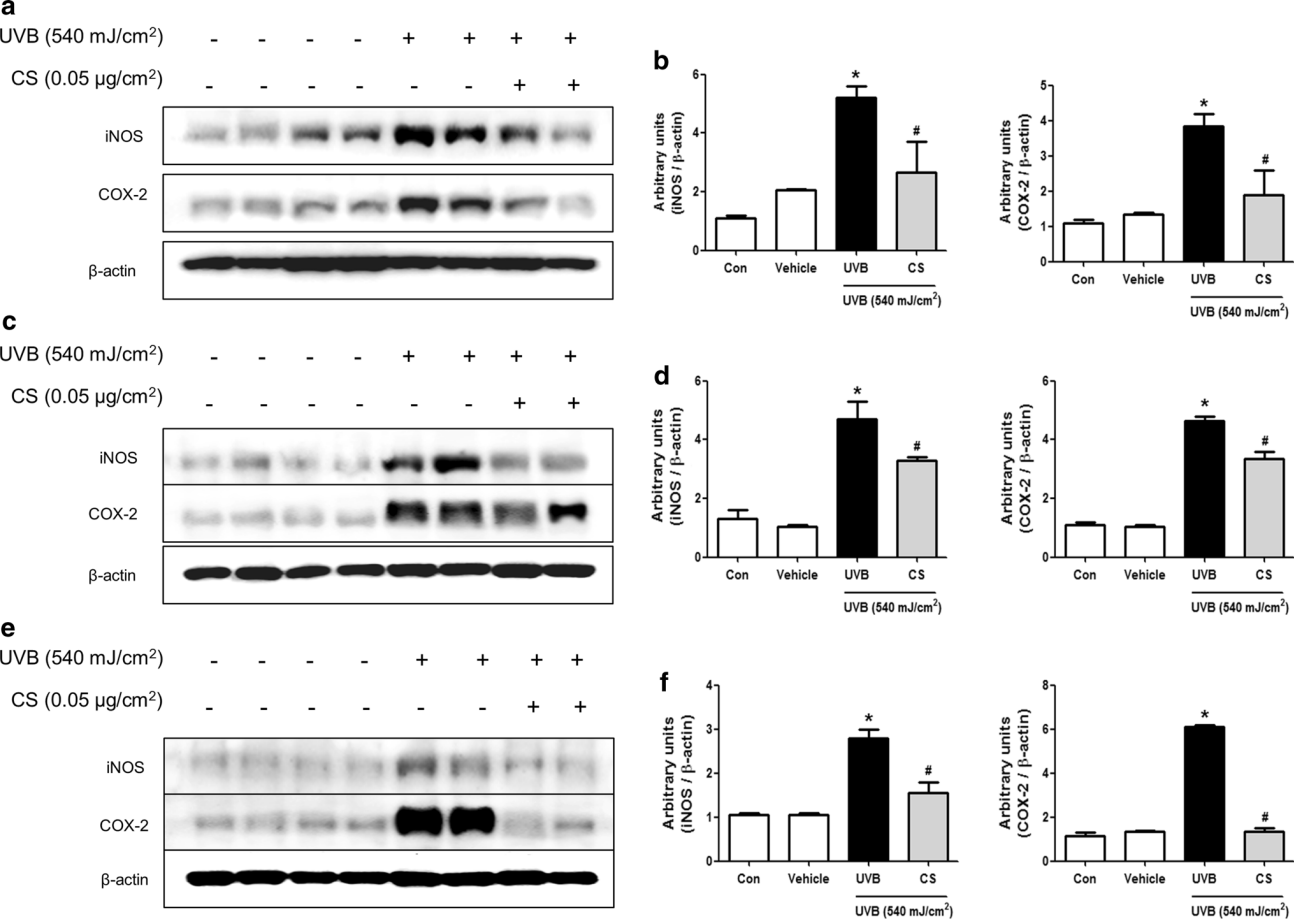
Expression level of iNOS and COX-2 protein in lymph node and ear or back skin. Protein expression levels of iNOS and COX-2 in lymph node (**a**), ear skin (**c**), and back skin (**e**) were measured by Western blotting. Relative densities of protein bands were quantified (**b**, **d**, **f**) following the procedure described in Materials and methods. Equal amounts of total proteins (20 μg/lane) were subjected to 10% SDS-PAGE and expression levels of iNOS and COX-2 protein were detected by Western blotting using specific antibodies. β-actin protein was used as an internal control. Data shown are mean ± SD (n = 2). *P < 0.05, significant difference compared to the control group. ^#^*P *< 0.05, significantly different compared to UVB exposed group

Lymph node is the first organ that produces inflammatory responses against the development of skin inflammation. We investigated whether carnosol could suppress inflammatory responses in lymph node by Western blot analysis. Protein expression levels of iNOS and COX-2 in lymph nodes were significantly upregulated in UVB exposed mice. However, such upregulated expression was significantly suppressed by carnosol treatment (Fig. 3a). Quantified relative densities of protein bands also showed significant suppressive effect of carnosol shown in Fig. 3b.

### Effect of carnosol treatment on inflammatory responses in ear and back

We also investigated whether carnosol could suppress inflammatory responses in ear and back skin tissues by Western blot analysis. Expression levels of iNOS and COX-2 in ear and back skin tissues were significantly increased in UVB exposed mice whereas carnosol treatment prevented such increase in the expression of iNOS and COX-2 (Fig. 3c–f).

### Effect of carnosol treatment on phosphorylation of STAT3 and JAK in ear and back

**Fig. 4:**
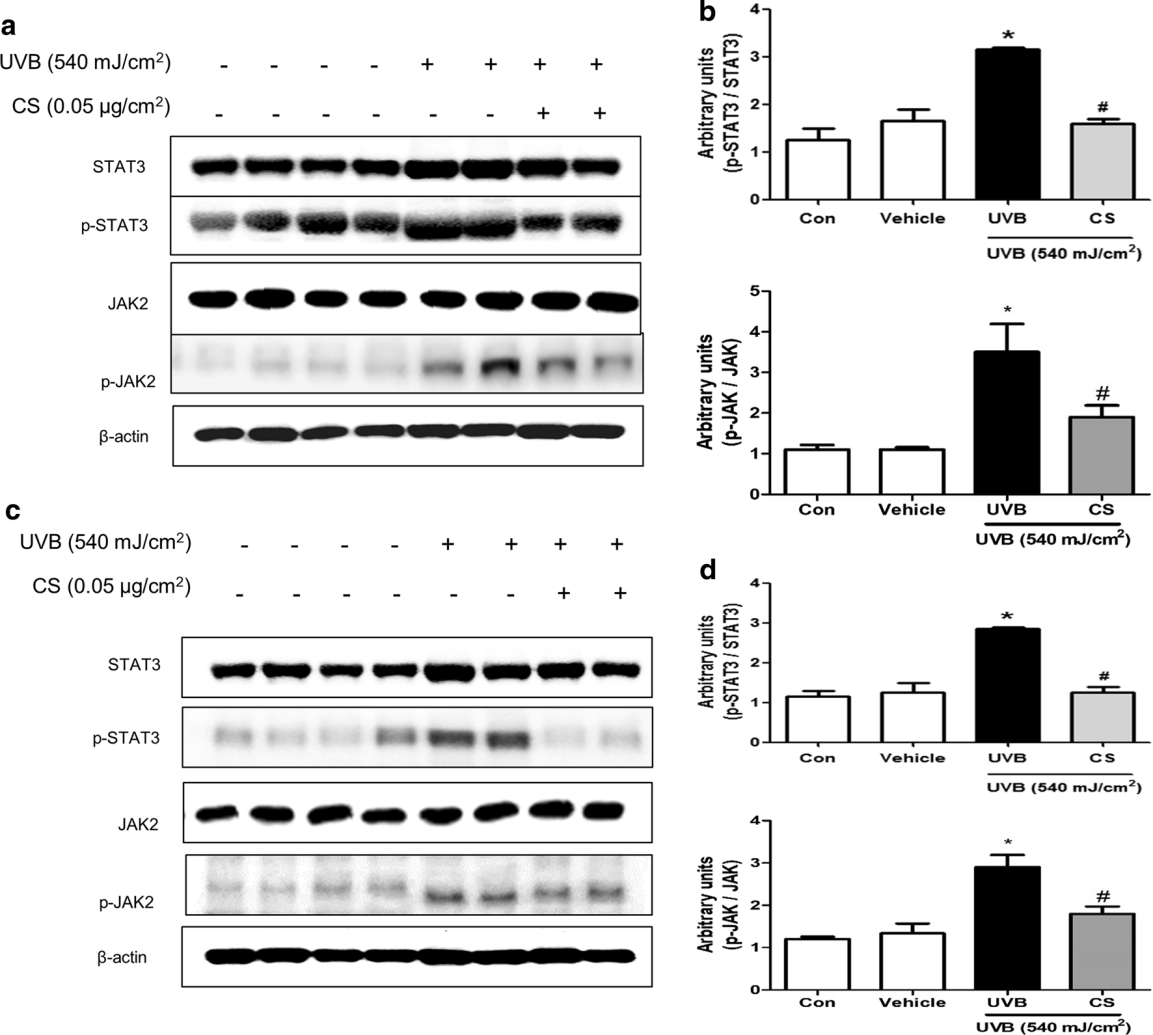
Expression level of STAT3 and p-STAT3 protein in ear or back skin. Protein expression levels of STAT3 and p-STAT3 in ear skin (**a**) and back skin (**c**) were measured by Western blotting. Relative densities of protein bands were quantified (**b**, **d**) following the procedure described in Materials and methods. Equal amounts of total proteins (20 μg/lane) were subjected to 10% SDS-PAGE and expression levels of STAT3 and p-STAT3 proteins were detected by Western blotting using specific antibodies. β-actin protein was used as an internal control. Data shown are mean ± SD (n = 2). *P < 0.05, significant difference compared to the control group. ^#^*P *< 0.05, significantly different compared to UVB exposed group

STAT3 and it upstream target pathway JAK are critical for inflammatory responses. To investigate the effect of carnosol on activation of STAT3 and JAK, we performed Western blot analysis. Protein expression levels of p-STAT3 and p-JAK in ear and back skin tissues were significantly increased in UVB exposed mice. However, they were significantly suppressed by carnosol treatment in both ear skin (Fig. 4a, b) and back skin (Fig. 4c, d).

### Effect of carnosol treatment on DNA binding activity of STAT3 in ear and back

**Fig. 5:**
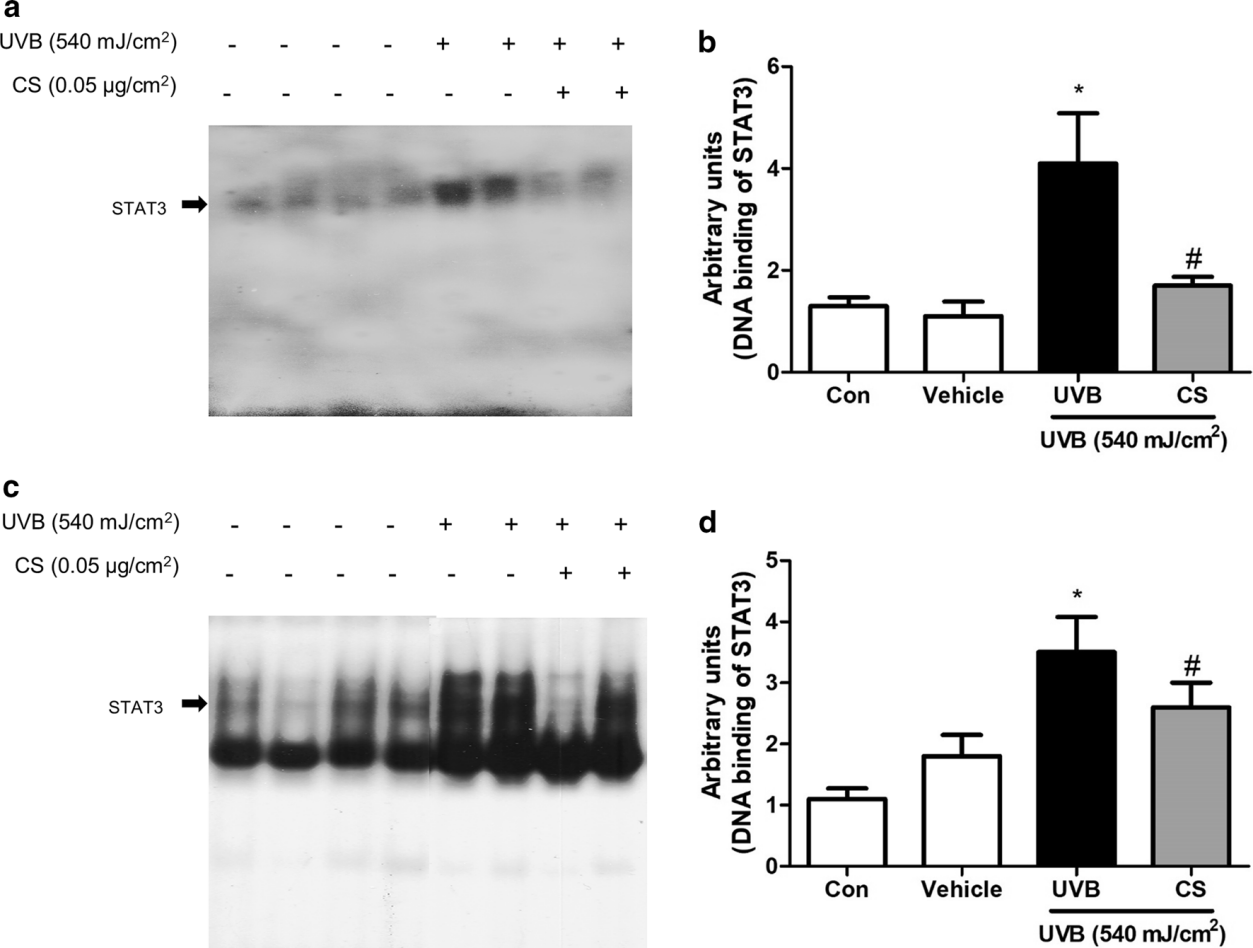
DNA binding actitiy of STAT3 in ear or back skin. DNA binding actitiy of STAT3 in ear skin (**a**) and back skin (**z**) were measured by EMSA, and densities of EMSA bands were quantified (**b**, **d**) following the procedure described in Materials and methods. Data shown are mean ± SD (n = 2). *P < 0.05, significant difference compared to the control group. ^#^*P *< 0.05, significantly different compared to UVB exposed group

We also determined the DNA binding activity of STAT3. Consistent with the inhibitory effect on STAT3 phosphorylation, DNA binding activity of STAT3 increased by the UVB exposure in both ear and skin tissues which was significantly reduced by carnosol in mice ear (Fig. 5a, c) and back skin (Fig. 5b, d).

## Discussion

Several studies have indicated that inflammatory skin disease such as atopic dermatitis is characterized by increased serum IgE level, upregulated pro-inflammatory genes, and increased release of cytokines (Choi et al. 2016). Our study demonstrated that carnosol treatment significantly reduced IgE, proinflammatory gene expression and cytokine releases in UVB-treated skin inflammation. It was reported that hypochlorous acid and tofacitinib downregulate IgE and cytokine level in a murine atopic dermatitis model (Fukuyama et al. 2018). It has been also reported that Hataedock, Quercetin, Resveratrol, and K112PC-5 possess anti-inflammatory effects by inhibiting NO production and expression of iNOS and COX-2 (Kang et al. 2008; Cichocki et al. 2008; Cha et al. 2016, 2017). These data indicating that carnosol could be useful for UVB-induced inflammatory skin damages.

Several studies have implicated that UVB exposure can suppress the immune system by activating STAT3 (Agilan et al. 2016). Activation of STAT3 and JAK can cause inflammatory and immunogenic skin damages by the release of TNF-α, IL-1β, and IL-10 and the increase of iNOS and COX-2 expression (Tyagi et al. 2012). It was reported that epidermal keratinocytes induce skin inflammation in the STAT3 overexpressed transgenic mice (Kumari et al. 2013). It was also reported that LPS-induced production of inflammatory cytokines can be completely blocked by STAT3-deficient macrophages (Takeda et al., 1999). Moreover, STAT3 mutation induced lower monocyte chemoattractant in adult patients with hyper-IgE syndrome. Thus, STAT3 inhibiting compounds could be applicable for inflammatory or immune mediated skin damages (Koppes et al. 2016; Park et al. 2017). STA-21, a promising STAT3 inhibitor that equally regulates Th17 and Treg cells, is known to prevent psoriasis and improve autoimmune inflammation (Miyoshi et al. 2011). In addition, caffeic acid can suppress chronic UVB-induced dermatitis by inhibiting STAT3 (Agilan et al. 2016). Our data showed carnosol inhibited UVB-induced STAT3 activation and its upstream signal JAK pathway. Suppressed STAT3 and JAK activation by carnosol was associated with reduced IgE level and atopic dermatitic skin conditions. Our previous study has shown that carnosol has significant STAT3 binding affinity (− 7.7 kcal/mol) (Lee et al. 2017). These results suggest that inhibition of inflammatory skin damages by carnosol could be mediated by STAT3 inactivation.

It has been reported that UVB exposure increases the expression of p-STAT3 in mouse skin (Chilampalli et al. 2011). Moreover, UVB-mediated epidermal hyperproliferation and regeneration are decreased in STAT3-deficient mice (Kim et al. 2009). These findings imply that STAT3 is indeed a novel potential target to prevent UVB radiation-induced inflammatory skin damages. Previous report has demonstrated that tannic acid prevents UVB-induced STAT3 expression in human retinal pigment epithelium cells (Chou et al. 2012). It was also reported topical or dietary silibinin can strongly protect against UVB exposure by down-regulating inflammatory and angiogenic responses through inhibition of STAT3 phosphorylation (Gu et al. 2007). It has been reported that carnosic acid isolated from rosemary (*Rosmarinus officinalis*) can inhibit UVB-induced ROS generation and expression of matrix metalloproteinases in human skin (Park et al. 2013). In addition, a protective effect of rosemary extract on contact dermatitis has been reported (Fuchs et al. 2005). In the present study, we demonstrated that carnosol also has protective effect on the UVB-induced inflammatory skin damages. We previously also reported that carnosol protected phthalic anhydride-induced inflammatory skin damages (Lee et al. 2017). Thus, our present data suggest that carnosol could be a therapeutic agent for inflammatory skin damages such as atopic dermatitis.
